# Eradication rates, risk factors, and implant selection in two-stage revision knee arthroplasty: a mid-term follow-up study

**DOI:** 10.1186/s13018-016-0428-4

**Published:** 2016-08-26

**Authors:** Steffen Hoell, Anna Sieweke, Georg Gosheger, Jendrik Hardes, Ralf Dieckmann, Helmut Ahrens, Arne Streitbuerger

**Affiliations:** 1 Department of Orthopaedics, Paracelsus Hospital, Am Natruper Holz 69, 49076 Osnabrück, Germany; 2 Department of General Orthopaedics and Tumor Orthopaedics, University Hospital, Albert-Schweitzer-Campus 1, 48149 Münster, Germany

**Keywords:** Two-stage revision knee arthroplasty, Arthrodesis, Risk factor, Periprosthetic joint infection, BMI, Nicotine abuse

## Abstract

**Background:**

Two-stage revision (TSR) knee arthroplasty is an established treatment, but failure to control infection still occurs in 4–50 % of cases. The aim of this study was to assess the infection eradication rate, risk factors for failure, and the clinical outcome after two-stage revision knee arthroplasty.

**Methods:**

This retrospective study included 59 patients who had undergone at least one two-stage revision procedure due to periprosthetic joint infection (PJI). Demographic data, comorbidities, types of implant, and complications were analyzed. Univariate and multivariate logistic regression analysis were used to identify risk factors for failure.

**Results:**

The infections were controlled in 55 patients (93.2 %). The follow-up period was 4.1 (±2.7) years. Infection control was achieved after the first TSR in 42 patients (71.2 %) and after the second TSR in 13 (76.5 %). The percentage of arthrodesis procedures in patients with infection control increased from 16.75 % after one TSR to 69.2 % after two TSRs. Multivariate logistic regression analysis identified body mass index (BMI) (odds ratio 1.22; 95 % confidence intervals, 1.07 to 1.40; *p* = 0.004) and smoking (OR 21.52; 95 % CI, 2.60 to 178.19; *p* = 0.004) as risk factors for failure.

**Conclusions:**

Two-stage revision protocols can achieve acceptable results even after a second procedure. It is still unclear whether the choice of implant influences failure rates. Risk factors for failure after two-stage revision were identified. Studies with larger sample sizes are needed in order to support these findings and identify further risk factors. To reduce failure rates, programs should be established to treat or minimize risk factors in patients with PJI.

## Background

Several studies have identified comorbidities and conditions that increase the rate of periprosthetic joint infection (PJI) after primary hip and knee arthroplasty [1–8]. In two-stage revision (TSR) surgery, protocols involving the implantation of an antibiotic-loaded bone–cement spacer have become the gold standard for treating periprosthetic infections.

Radical debridement with explantation of the prosthesis and supportive administration of antibiotics are the most important pillars for controlling PJI [9, 10], but reinfection rates after TSR continue to be high. Reinfection rates reported in the literature range from 4 to 50 % [3, 4, 11–16]. Only a few studies have analyzed the factors that have a negative impact on infection control after TSR [17–20]. In order to minimize failure rates in TSR, evaluated treatment protocols and diagnostic algorithms are needed, and it should be possible to identify patients who are at higher risk of failure. Once risk factors have been identified, further investigations and additional treatments can help reduce failure rates.

The aim of the present study was to investigate the extent to which infection can be successfully controlled after two-stage revision knee arthroplasty and identify factors that influence the failure rate.

## Methods

Seventy patients who underwent two-stage revision knee arthroplasty between 2004 and 2008 in our department were identified. The following criteria were used to define PJI: sinus tract communicating with the prosthesis and/or at least two identical positive cultures identified intraoperatively [21, 22]. All infections were defined as delayed or late chronic [23].

Seven patients had died and four patients declined to participate in the study, and a total of 59 patients were therefore included. Their average age at follow-up was 73 years (±9.7), and there were 32 men and 27 women. The patients were all referred to our institution as a tertiary center. The protocol consisted of explantation of the prosthesis with implantation of a fixed antibiotic-loaded bone–cement spacer (Refobacin® Revision bone cement; Biomet Inc., Warsaw, Indiana, USA; 1 g gentamicin and 1 g clindamycin/40 g cement) and at least 14 days of intravenous antibiotic administration, followed by at least 4 weeks of antibiotics orally. If necessary, additional antibiotics were mixed into the spacer, depending on the microbiological results, as an off-label application. All antibiotic treatments were administered in collaboration with the hospital’s Institute of Microbiology. After an interval of 14 days without antibiotics, C-reactive protein (CRP) was measured in serum. The second-stage procedure was performed 9–12 weeks after explantation.

The criteria for reimplantation were no sinus track, no signs of local inflammation, and serum CRP values that had declined during the period since explantation. The definition of infection control was no subsequent surgical intervention for infection at the time of follow-up. Eleven potential risk factors were documented from the demographic data, comorbidities, and postoperative complications.

The criteria for arthrodesis (*n* = 18 patients) were an insufficient extension mechanism and/or clearly compromised capsule and soft-tissue conditions, with a high risk of postoperative wound healing problems and limited function. The indication for arthrodesis was based on the personal judgment and experience of the surgeon and the patient’s consent. None of the patients underwent additional soft-tissue coverage with local muscle flaps.

### Statistical analysis

Means plus or minus standard deviation (SD), ranges, and proportions were calculated to analyze the different characteristics in the cases of two-stage knee revision. Statistical significance was assessed using the chi-squared test, Fisher’s exact test, Student’s *t* test, and the Mann-Whitney *U* test.

The probability of failed infection control was modeled using univariate binary logistic regression. Odds ratios, the corresponding 95 % confidence intervals, and Wald-type *p* values were calculated. In a second step, variables were selected in a stepwise fashion, applying backward selection to variables in the univariate logistic regression. All inferential statistics are intended to be exploratory, not confirmatory, and are interpreted accordingly. The comparison-wise type 1 error rate is controlled instead of the experiment-wise error rate. The local significance level was set to 0.05.

No adjustment for multiple testing was performed. Statistical analyses were performed using IBM SPSS® Statistics for Windows, version 21 (IBM Corporation, Armonk, NY, USA).

## Results

Infection control was achieved in 55 patients (93.2 %). The follow-up period was 4.1 years (±2.7 years). Infection control was achieved after the first TSR in 42 patients (71.2 %) and after the second TSR in 13 patients (76.5 %). There were no significant differences between the first and second TSRs (*p* > 0.05). The percentage of arthrodesis in patients with infection control increased from 16.75 % after one TSR to 69.2 % after two TSRs. The average time from reimplantation to reinfection was 2.3 years (range 0.6–3.7 years).

**Fig. 1:**
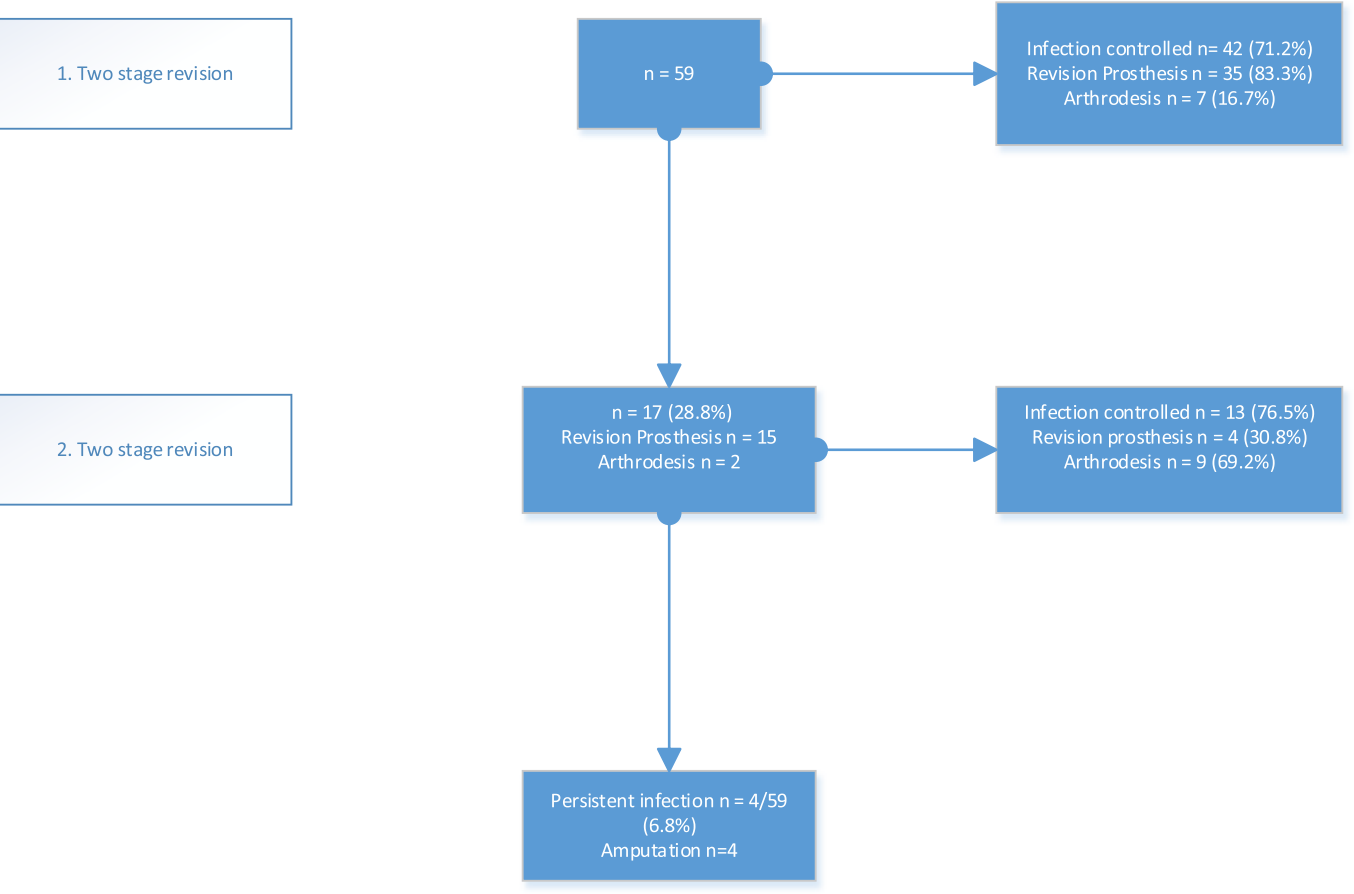
Flowchart of all patients

The amputation rate when infection could not be controlled was 6.8 % (4/59); amputations were required in one patient with an arthrodesis and three with revision endoprostheses. Figure 1 shows the clinical course for all of the patients. The risk factors investigated and the results of the univariate logistic regression are listed in Table 1. Although patients who had *Staphylococcus epidermidis* at the first revision had the highest failure rate (35.3 %), statistical analysis was not performed due to the small number of cases. Table 2 presents the results of the multivariate logistic regression analysis after variable selection. Table 3 shows the organisms that were cultured in patients with recurrent infections and the choice of implant. Identical bacteria were found at the second TSR in eight of the 17 patients concerned (47.1 %).

**Table 1:** Potential risk factors for faiure that were investigated with univariable logistic regression

		Infection controlled after the first TSR	Fialure after the first TSR	*P* value	Odds ratio	CI (95 %)
Sinus present				*0.008*	5.24	1.55–17.65
Yes	*n* = 19	*N* = 10	*N* = 9			
No	*n* = 40	*N* = 35	*N* = 5			
Diabetes				*0.009*	6.65	1.62–27.3
Yes	*n* = 11	*n* = 4	*n* = 7			
No	*n* = 48	*n* = 38	*n* = 10			
Smoking				*0.018*	8.33	1.43–48.54
Yes	*n* = 7	*N* = 3	*N* = 4			
No	*n* = 52	*N* = 42	*N* = 10			
BMI >30				*0.033*	5.74	1.15–28.62
Yes	*n* = 37	*n* = 24	*n* = 13			
No	*n* = 22	*n* = 17	*n* = 5			
Periprosthetic fracture				*0.034*	3.57	1.1–11.57
Yes	*n* = 23	*n* = 14	*N* = 9			
No	*n* = 36	*n* = 28	*N* = 8			
Wound healing problems				0.061	3.16	0.95–10.55
Yes	*n* = 17	*N* = 10	*N* = 7			
No	*n* = 42	*N* = 35	*N* = 7			
Corticosteriods				0.076	8.38	0.8–87.11
Yes	*n* = 4	*N* = 2	*N* = 2			
No	*n* = 55	*N* = 43	*N* = 12			
Immune suppression				0.191	5.2	0.44–61.67
Yes	*N* = 1	*N* = 2				
No	*N* = 45	*N* = 11				
Postoperative hematoma				0.418	1.67	0.48–5.8
Yes	*n* = 16	*N* = 11	*N* = 5			
No	*n* = 43	*N* = 29	*N* = 14			
Blood transfusion				0.458	2.37	0.24–23.1
Yes	*n* = 44	*N* = 28	*N* = 16			
No	*n* = 15	*N* = 14	*N* = 1			
Tumor disease				0.986	1.02	0.18–5.91
Yes	*n* = 7	*N* = 5	*N* = 2			
No	*n* = 52	*N* = 40	*N* = 12			

*P* value, significance level was set to 0.05

**Table 2:** Comorbid conditions or patterns that were identified by variable selection as risk factors in a multivariable logistic regression

	*P*	Odds ratio	CI (95 %)
Body mass index (kg/m^2^)	0.004	1.22	1.07–1.40
Nicotine abuse	0.004	21.52	2.60–178.19

**Table 3:** Patients with recurrent infection

Patients	Culture during the first TSR	Culture during the second TSR	Implant after the first TSR	Outcome after the second TSR
1	Staph aureus	Staph aureus	Revision prosthesis	Revision prosthesis
2	Staph aureus	Staph aureus	Revision prosthesis	Revision prosthesis
3	Staph aureus	Staph aureus	Revision prosthesis	Revision prosthesis
4	Staph epi	Staph epi	Revision prosthesis	Arthrodesis
5	Staph epi	Staph epi	Revision prosthesis	Arthrodesis
6	Staph epi	Staph epi	Revision prosthesis	arthrodesis
7	Staph epi	Staph epi	Revision prosthesis	Revision prosthesis
8	Staph capitis	Staph capitis	Revision prosthesis	Arthrodesis
9	Staph epi	Staph haemolyticus	Revision prosthesis	Arthrodesis
10	Staph epi	MRSA	Revision prosthesis	Arthrodesis
11	Staph epi	MRSA	Revision prosthesis	Amputation
12	Staph epi	MRSA	Revision prosthesis	amputation
13	Enterobacter faecalis	E. coli	Revision prosthesis	Arthrodesis
14	Staph aureus	Staph epi	Revision prosthesis	amputation
15	Staph simulans	Staph epi	Revision prosthesis	Arthrodesis
16	Staphylococcus hominis Streptococcus acidominimus	Candida albicans	arthrodesis	amputation
17	Staphylococcus epidermidis/Klebsiella oxytoca/Pseudomonas aeruginosa/Enterococcus faecalis	Candida albicans	Revision prosthesis	Arthrodesis

## Discussion

Periprosthetic joint infection (PJI) is one of the most severe complications that occur in patients who undergo total knee arthroplasty (TKA). Two-stage revision is still the gold standard for treatment of PJI, although one-stage revisions may achieve similar results in special conditions [24–28]. Nevertheless, reinfection rates vary from 4 to 50 % [3, 4, 11–16]. Among the patients included in the present study, successful treatment was achieved in 55 (93.2 %) after a mean follow-up period of 4.1 years.

There were no differences in the success rates between patients who underwent one TSR procedure and those with two procedures. Lower eradication rates have been reported in the literature after a second TSR [29], but a high rate of arthrodesis in the second TSRs might be an explanation for this. Isiklar et al. recommended arthrodesis instead of multiple revisions in patients with chronic infections, in order to avoid amputation [15].

Other studies have also reported higher rates of infection control with arthrodesis in comparison with revision prostheses [2, 11, 16, 30]. In contrast to these results, a 50 % failure rate after septic arthrodesis was reported in 2015 [31].

In view of the small numbers of arthrodeses, statistical analysis was not carried out in the present study and no conclusions can therefore be drawn on whether or not arthrodesis is in fact associated with lower reinfection rates.

It has to be discussed if allograft reconstruction of the extensor mechanism is an alternative instead of arthrodesis. Although it is known that allograft reconstructions show high rates of complications the benefit of a better mobility must be considered. In a study from 2016 in 26 knees, 69 % of the allografts could be retained at a follow-up of 68 months with a reoperation rate of 58 % [32, 33].

However, it is not only the type of treatment administered that is responsible for the clinical outcome. It is known from several studies that comorbidities and other conditions can have a negative influence on infection rates after primary arthroplasty [1–8]. The causes of failure after TSR are rarely reported [17–20].

The most frequent potential risk factors for failure were analyzed in the patients included in the present study. Among the comorbid conditions present, diabetes was identified as a risk factor, with an OR of 6.65 (95 % CI, 1.62 to 27.30) in the univariate analysis. Another study published in 2015 also found that diabetes had a significantly higher prevalence in the group with reinfections [19]. By contrast, Sakellariou et al. did not find any significant differences in a univariate analysis of 110 patients with TSR [18]. Among the local conditions that were present, fistulas were found to be a relevant factor in the univariate analysis. This finding is supported by a study also published in 2015, in which fistulas were associated with recurrent infection even in the multivariate logistic regression analysis [20].

A medical history including periprosthetic fracture around the knee was identified as a risk factor for failure after the first TSR. In an earlier study, our group showed that septic failure of revision arthroplasty with megaprostheses was strongly associated with a medical history of periprosthetic fractures around the knee [13].

Suzuki et al. investigated the influence of surgical procedures in the region of the knee joint. They observed significantly more frequent infections with open reduction and internal fixation after trauma to the knee joint and when osteosynthesis material remained in situ [7].

Two risk factors were identified in the multivariate logistic regression analysis in the present study: body mass index (BMI) and smoking. An increase in the BMI by one point showed an increased risk for failure of about 22 %. However, Mortazavi et al. did not observe any association between BMI and persistent PJI after two-stage TKA [25]. Kubista et al. distinguished between BMI scores of <25, 25–35, and >35.

No significant differences were observed between these groups with regard to the rates of persistent infection after two-stage TKA [34]. In a matched-cohort study, patients with a BMI >40 kg/m^2^ had a 22 % risk for reinfection in comparison with patients with a BMI <30 kg/m^2^, at 4 % [35]. In two-stage revision hip arthroplasty, obesity has also been found to be a significant risk factor for failure [36]. Higher rates of recurrent infection have also been reported among smokers, with a 71.4 % rate of persistent or recurrent infection after the first two-stage replacement in comparison with only 23.1 % in nonsmokers [6, 7, 37]. These results were confirmed in the present cohort.

The study has several limitations. As all of the patients were referred to the department, it was not possible to record all relevant factors. For example, the number of previous revision procedures was unclear and could not be analyzed, although it is known that this factor has a negative influence on complication rates [13, 17]. Due to the relatively small number of patients who underwent arthrodesis, statistical analysis was not useful. The wide variety of bacteria identified also made it impossible to carry out statistical analysis.

## Conclusions

Two-stage revision (TSR) protocols can achieve acceptable results even when they are repeated. Amputation rates can be kept low. It is still unclear whether the choice of implant has an influence on failure rates. Risk factors for failure after two-stage revision have been identified, but studies with larger numbers of patients are needed in order to support these findings and identify further risk factors. In order to reduce failure rates, programs should be established for treating or minimizing risk factors in patients with periprosthetic joint infection.

## Abbreviations

BMI: Body mass index
CI: Confidence interval
CRP: C-reactive protein
OR: Odds ratio
PJI: Periprosthetic joint infection
SD: Standard deviation
TKA: Total knee arthroplasty
TSR: Two-stage revision
